# Lifespan, Healthspan, and the Expanding Role of Cosmetic Dermatology in Longevity Science

**DOI:** 10.1111/jocd.70610

**Published:** 2025-12-15

**Authors:** Diala Haykal, Frederic Flament, Khaled Othman, Richard Siow

**Affiliations:** ^1^ Centre Laser Palaiseau Private Practice Palaiseau France; ^2^ L'Oréal Research and Innovation Clichy France; ^3^ Chairman of Skin and Slim Medical Center, Certified Consultant Dermatology, Aesthetic Medicine, and Andrology Abu Dhabi UAE; ^4^ Ageing Research at King's (ARK), Faculty of Life Sciences & Medicine King's College London London UK

To the Editor,

The growing distinction between lifespan, the total years lived, and healthspan, the years lived in good health, free from chronic disease and functional decline, has reshaped the priorities of longevity science. Within this evolving paradigm, cosmetic dermatology is emerging as more than a discipline of aesthetic refinement; it is becoming a meaningful participant in promoting healthspan and contributing to broader longevity strategies [1].

Traditionally, cosmetic dermatology has addressed visible signs of skin aging such as wrinkles, laxity, pigmentation, and volume loss. These manifestations, once regarded purely as aesthetic concerns, increasingly reflect deeper biological processes including oxidative stress, chronic inflammation, cellular senescence, and extracellular matrix degradation. As such, the skin functions both as a mirror of systemic aging and as a potential lever for intervention. Emerging treatments, including energy‐based devices, biostimulatory injectables (such as poly‐L‐lactic acid), microneedling radiofrequency, and barrier‐ or microbiome‐supporting topicals, demonstrate promise in modulating these biological processes, thus aligning aesthetic care with tissue longevity and regenerative goals [2].

However, the dermatologic contribution to healthspan extends beyond aesthetic aging. Chronic inflammatory skin conditions such as acne, rosacea, psoriasis, and atopic dermatitis also shape both the physiological and psychosocial dimensions of aging. These diseases are associated with systemic inflammation, immune dysregulation, metabolic disturbance, and psychological burden, all of which accelerate biological aging and impair quality of life [3, 4, 5]. Their management, therefore, represents a vital component of longevity‐oriented dermatologic care. Integrating anti‐inflammatory, barrier‐restoring, and microbiome‐balancing therapies not only improves cutaneous health and appearance but may also mitigate “inflammaging,” reduce oxidative stress, and restore homeostasis across organ systems [6, 7].

The advent of cutaneous biomarkers and skin‐based epigenetic clocks further positions dermatology within the domain of longevity science. These tools offer non‐invasive means to assess biological age, monitor individual aging trajectories, and evaluate response to anti‐aging interventions, supporting a shift from reactive to preventive and personalized care. Beyond physiology, the psychosocial benefits of cosmetic interventions, improvements in self‐esteem, reductions in anxiety and depression, enhanced social engagement are increasingly recognized as integral to healthy aging. These outcomes, while subjective, correlate with positive health behaviors, reduced perceived stress, and improved resilience, thus contributing to extended healthspan. Recent research has further revealed links between the skin microbiome and psychological wellbeing, suggesting that interventions aimed at supporting a healthy skin ecosystem may not only improve cutaneous health but also promote mental health and emotional resilience [8].

To realize the full potential of cosmetic dermatology in longevity medicine, we advocate for a reorientation of practice: from episodic treatments aimed at isolated concerns to comprehensive, longitudinal strategies that preserve both aesthetic and functional skin health. This framework should integrate the management of chronic dermatoses with regenerative and preventive interventions to optimize skin and systemic well‐being. Multidisciplinary collaboration, enhanced patient education, and clinician training in aging biology and regenerative science will be essential [9].

In this evolving paradigm, cosmetic dermatology is uniquely positioned to help redefine beauty, not as a superficial or purely aesthetic pursuit, but as a visible expression of biological harmony, resilience, and vitality. By embracing an integrative approach that unites regenerative science, chronic skin health, and psychosocial well‐being, the field transcends traditional boundaries to become an active contributor to human longevity. By aligning with preventive and personalized strategies, cosmetic dermatology can meaningfully extend healthspan, ensuring that longer lives are accompanied by healthier, more functional, and confident years. Through regenerative interventions, holistic care, and interdisciplinary collaboration, our specialty affirms its role not merely as a provider of visual enhancement, but as a partner in sustaining well‐being, dignity, and quality of life across the lifespan [10] (Figure 1).

**FIGURE 1:**
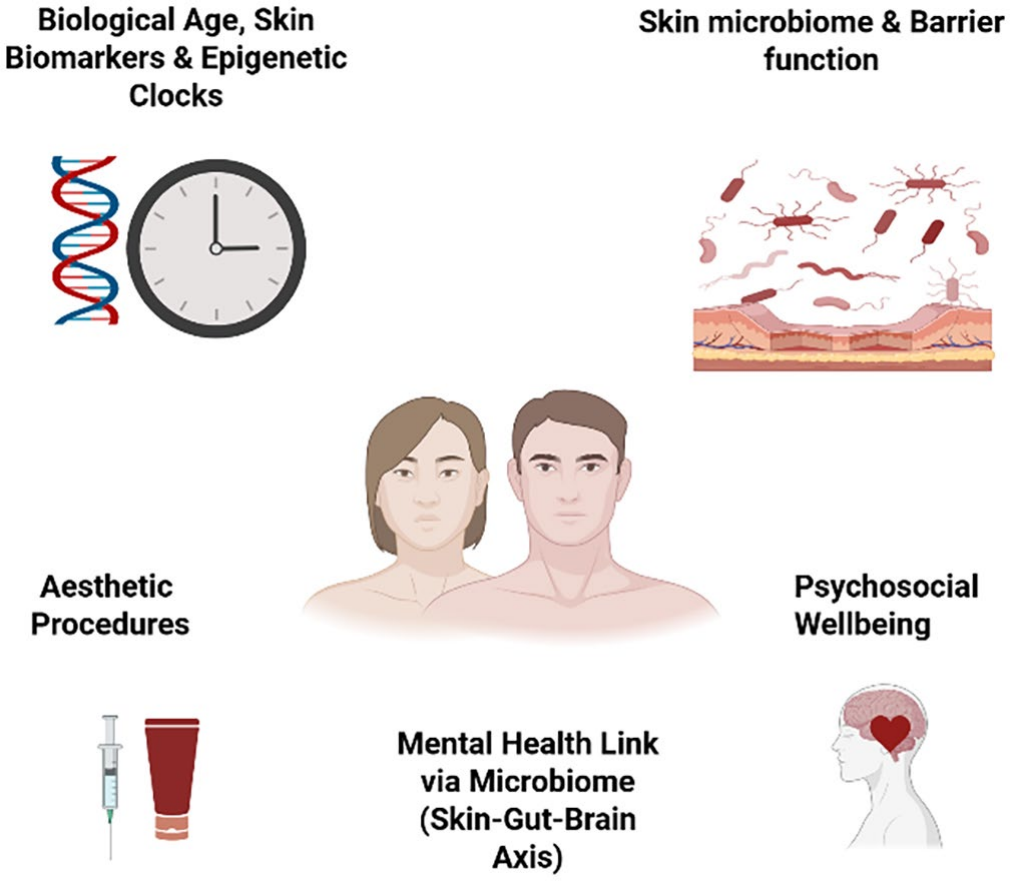
Integrative cosmetic dermatology in healthspan and longevity.

## Ethics Statement

The authors have nothing to report.

## Consent

The authors have nothing to report.

## Conflicts of Interest

The authors declare no conflicts of interest.

## Data Availability

The data that support the findings of this study are available on the references' part.
